# Co-morbid Indomethacin-Responsive Headaches in a Woman in Her Late 60s With Paroxysmal Hemicrania and Hypnic Headache: A Case Report

**DOI:** 10.7759/cureus.77243

**Published:** 2025-01-10

**Authors:** Ashlyn Brown, Randolph W Evans, Claudia Carrizo, Mark Burish

**Affiliations:** 1 Physical Medicine and Rehabilitation, Baylor College of Medicine, Houston, USA; 2 Neurology, Baylor College of Medicine, Houston, USA; 3 Neurosurgery, University of Texas Health Science Center at Houston, Houston, USA

**Keywords:** headaches disorder, headache treatment, hypnic headache, indomethacin-responsive headaches, indomethacin therapy, paroxysmal hemicrania, trigeminal autonomic cephalalgia

## Abstract

Paroxysmal hemicrania (PH) and hypnic headache (HH) are rare indomethacin-responsive headache syndromes. This case report details the new onset of both disorders in a woman in her late 60s. One headache type presented as severe pain centered on the right eyebrow, lasting 30 minutes, occurring more than 8 times daily, and associated with ipsilateral lacrimation and rhinorrhea. The second type was a right frontal severe pain, with onset at 4 a.m., occurring only during sleep, lasting 30 minutes, and with no associated factors. The patient's response to indomethacin for both headache types was confirmed through an unblinded ABAB study design: complete resolution of headaches during indomethacin use and recurrence upon discontinuation. This case highlights the rarity of co-morbid indomethacin-responsive headaches and underscores the diagnostic and therapeutic challenges associated with these conditions.

## Introduction

Paroxysmal hemicrania (PH) and hypnic headache (HH) are rare, indomethacin-responsive headache syndromes, each with distinct clinical presentations [1]. PH, first described in 1974 by Sjaastad and Dale [2], is defined as attacks of severe unilateral pain in or around the eye, lasting 2-30 minutes and occurring at least six times per day, associated with ipsilateral cranial autonomic features (such as lacrimation) and/or restlessness. An important criterion for the official diagnosis is that it can be absolutely prevented by indomethacin [1]. PH is one of five headaches collectively referred to as trigeminal autonomic cephalalgias, reflecting the trigeminal location of pain and involvement of cranial autonomic pathways. The other trigeminal autonomic cephalalgias include hemicrania continua, cluster headache, short-lasting unilateral neuralgiform headache attacks with conjunctival injection and tearing (SUNCT), and short-lasting unilateral neuralgiform headache attacks with cranial autonomic symptoms (SUNA). PH and hemicrania continua are indomethacin-responsive, while cluster headache, SUNCT, and SUNA are not. PH has not been extensively researched but has a suspected prevalence of 1 in 2000, a typical onset around 40 years old, and a slight female predominance [3,4].

HH, also known as "alarm clock headache," was first described by Raskin in 1988 and is defined by attacks occurring only during sleep (and waking the patient), lasting 15-240 minutes, occurring at least 10 days per month, and having no associated cranial autonomic symptoms or restlessness [1,5]. While HH is not a trigeminal autonomic cephalalgia, it shares three overlapping features with the trigeminal autonomic cephalalgias. First, like PH and hemicrania continua, HH responds well to indomethacin, which is considered a third-line HH treatment behind caffeine and lithium [6]. Second, HH was previously called "alarm clock headache" due to its predictable occurrence at the same time each night; cluster headache shares a similar clocklike regularity in its attacks [7]. Finally, HH attacks last 15-240 minutes, overlapping in duration with both PH (2-30 minutes) and cluster headache (15-180 minutes). Though its prevalence remains uncertain, HH has a female predominance of 2:1 and primarily affects individuals over 50, although cases have been reported in younger individuals [8-10].

This case report focuses on a patient with both a PH-type headache and an HH-type headache. Both headache types were entirely prevented with indomethacin. We explore her clinical presentation, work-up, and treatments (including the ineffectiveness of all non-indomethacin treatments and the all-too-common tolerability issues with indomethacin). These diseases, though rare, underscore the importance of clinical vigilance and the profound therapeutic potential of indomethacin in managing specific headache syndromes.

## Case presentation

A 77-year-old female with a history of sacroiliac joint arthropathy and neck pain described three right-sided headache types (she has only experienced left-sided head pain in the context of sinus issues).

First headache type (“migraine-type”)

This headache first appeared when she was a teenager. It is a right-sided throbbing pain lasting approximately 4 hours, occurring 0-2 times per month, triggered by nitrates and monosodium glutamate, and treated effectively with sumatriptan. It is associated with ipsilateral nasal congestion, nausea, and vomiting but lacks photophobia or phonophobia. This headache disorder was well-managed with sumatriptan, and given its infrequent nature (0-2 times per month), no additional treatments were administered (Table 1).

**Table 1:** Official features of trigeminal autonomic cephalalgias and hypnic headache. SUNCT: Short-lasting unilateral neuralgiform headache attacks with conjunctival injection and tearing; SUNA: Short-lasting unilateral neuralgiform headache attacks with cranial autonomic symptoms.

	Paroxysmal hemicrania	Cluster headache	Hemicrania continua	SUNCT	SUNA	Hypnic headache
Location	Unilateral orbital, supraorbital and/or temporal	Unilateral orbital, supraorbital and/or temporal	Unilateral headache	Unilateral orbital, supraorbital, temporal and/or other trigeminal distribution	Same as SUNCT	No mention of location in criteria
Severity	Severe	Severe or very severe	Exacerbations of moderate or greater	Moderate or severe	Same as SUNCT	No mention of severity in criteria
Duration	2-30 minutes	15-180 minutes	>3 months	1-600 seconds	Same as SUNCT	15-240 minutes after waking
Frequency	>5 per day	every other day to 8 per day	constant for >3 months	At least 1 per day	Same as SUNCT	>9 days per month
Associated features	Cranial autonomic features and/or restlessness	Cranial autonomic features and/or restlessness	Cranial autonomic features and/or restlessness/worsening with movement	Conjunctival injection and lacrimation	Cranial autonomic features (except not both conjunctival injection and lacrimation)	Develop only during sleep No cranial autonomic features or restlessness
Indomethacin response	Prevented absolutely by indomethacin	No mention of indomethacin in criteria	Prevented absolutely by indomethacin	No mention of indomethacin in criteria	Same as SUNCT	No mention of indomethacin in criteria

Second headache type (“HH-type”)

This headache began in her late 60s, with no clear inciting event. It presents as a right frontal pain with an intensity of 7 out of 10, occurring once daily at 4 a.m. and never while awake. There are no associated factors with this headache type.

Third headache type (“PH-type”)

This headache started a few months after the “HH-type” headache, also without a clear inciting event. It is a right-sided pain with an intensity of 6-7 out of 10, centered on the eyebrow and radiating to the neck. It lasts approximately 30 minutes and occurs daily, about every 2.5 hours throughout the day and night, resulting in more than 8 attacks per day. It is associated with ipsilateral lacrimation and rhinorrhea but lacks restlessness, nausea, vomiting, photophobia, or phonophobia. One of the PH-type attacks reliably occurs at the same time daily (initially between 12-1 p.m., later shifting to the mid-afternoon).

**Figure 1:**
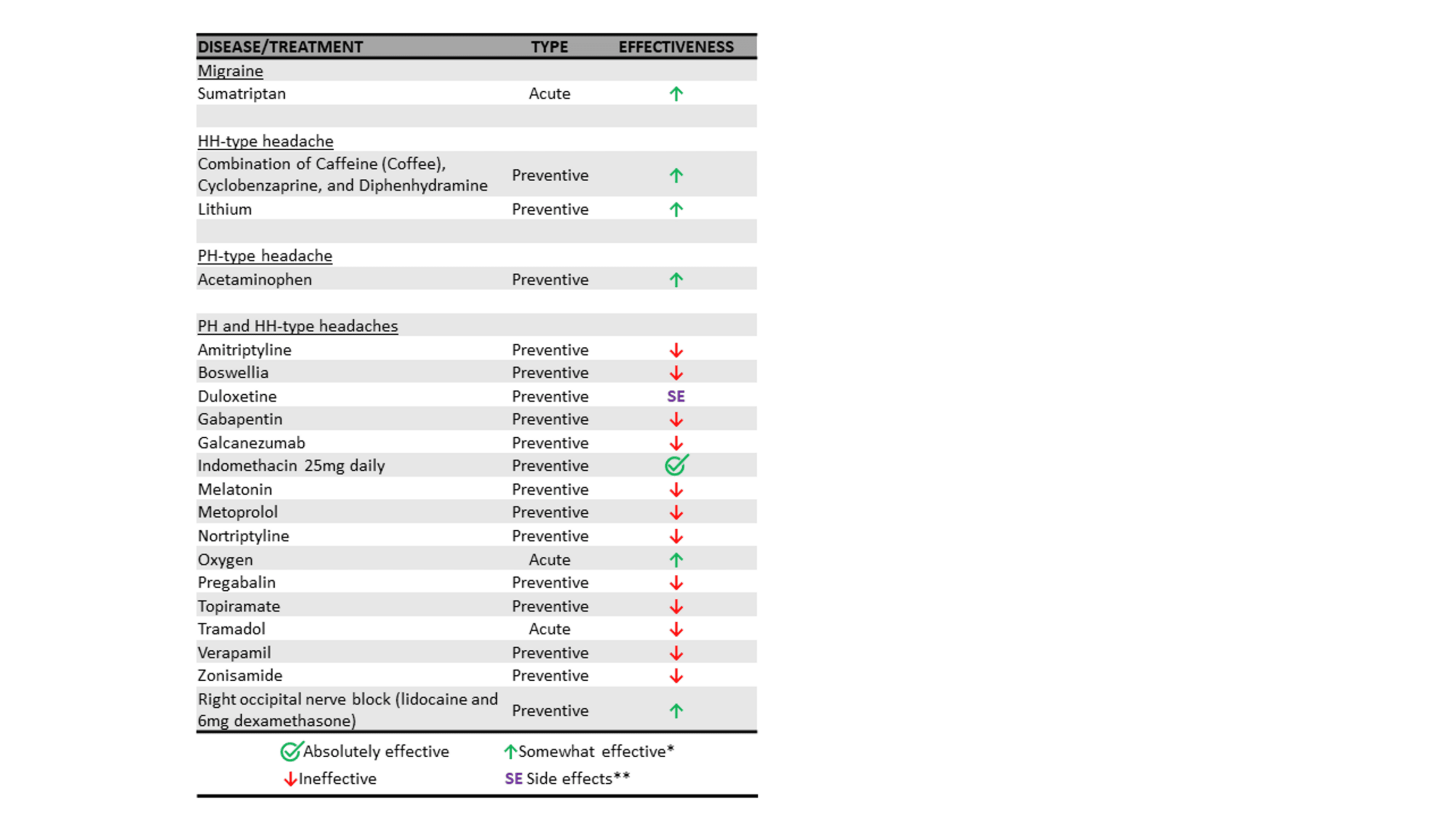
Summary of medications tried for the patient’s three headache types. *Somewhat effective medications explained: A combination of caffeine, cyclobenzaprine, and diphenhydramine was mildly effective for the HH-type headache if taken before bedtime; sumatriptan successfully aborted migraine attacks; lithium reduced HH-type attacks from 4 per day to 3 per day; acetaminophen was effective for the PH-type headache if taken at 3500-4000 mg divided four times daily; oxygen provided variable effectiveness; and an occipital nerve block gave temporary relief (less than 1 day). **Duloxetine was stopped quickly due to nausea and insomnia. HH: Hypnic headache; PH: Paroxysmal hemicrania.

For her second and third headache types, the patient tried various treatments, most of which were either ineffective or caused significant side effects (Figure 1).

Work-up

**Figure 2:**
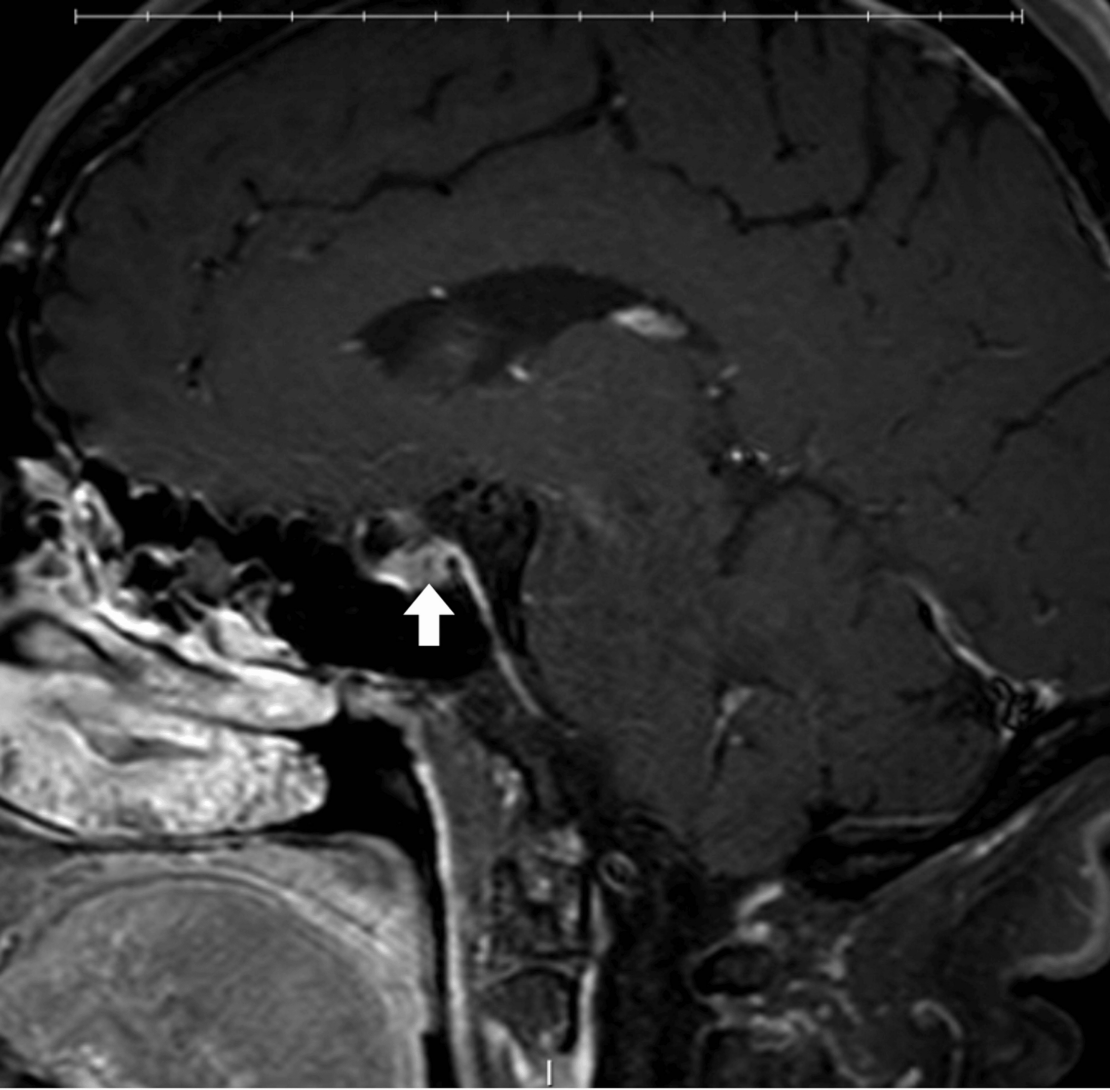
Contrast-enhanced T1-weighted fat-saturated brain MRI in the sagittal view shows a punctate focus of hypoenhancement (arrow) compared to the normally enhancing surrounding pituitary gland.

Neurological examination was unremarkable. A work-up for secondary causes of trigeminal autonomic cephalalgias included two brain MRIs, one conducted 4 years before the onset of her new headaches and one 3 years after. Both revealed a questionable pituitary adenoma (Figure 2) (the second MRI included thin cuts of the pituitary), as well as mild-to-moderate chronic microvascular ischemia, but were otherwise unremarkable. A pituitary panel (comprising thyroid-stimulating hormone, cortisol, prolactin, luteinizing hormone, follicle-stimulating hormone, adrenocorticotropic hormone, growth hormone, total testosterone, and total estrogen) was also unremarkable. MRA of the head and neck showed no abnormalities.

The patient underwent an indomethacin trial. Initially, her headaches were 90% controlled with indomethacin 25 mg daily and completely resolved with 25 mg twice daily. Subsequently, 25 mg once daily was sufficient for complete resolution of her headaches. We observed an unblinded ABAB-type study with indomethacin: the medication was temporarily stopped due to palpitations (later determined to be unrelated) and again a year later due to elevated creatinine (but was restarted after normalization of creatinine). Each time, while taking indomethacin 25 mg once daily, her PH-type and HH-type headaches resolved entirely (no attacks). In contrast, indomethacin had no effect on her migraine-type headaches (neither frequency nor other characteristics). When stopping indomethacin, both PH-type and HH-type headaches returned quickly. Upon restarting indomethacin, both headache types resolved again. The patient was last interviewed 3 years after restarting indomethacin and continues to experience complete resolution of her PH-type and HH-type headaches on indomethacin 25 mg daily. She also continues to have 0-2 migraine-type headaches per month, treatable with sumatriptan. While her creatinine has remained normal, she has developed mild hyperkalemia, possibly related to indomethacin, and is under the care of a nephrologist.

All three headaches met the International Classification of Headache Disorders (3rd edition) criteria for migraine, hypnic headache, and paroxysmal hemicrania, respectively [1]. We refer to hypnic headache and paroxysmal hemicrania as HH-type and PH-type, as they may be related (given their onset around the same time) or may represent secondary rather than primary headaches (as discussed below).

## Discussion

Our case illustrates the presentation of three headache types in the same patient: migraine in her youth and PH-type and HH-type headaches a few months apart in her 60s. We suspect that she also has migraine unrelated to her PH-type or HH-type headaches, both because migraine onset was approximately 50 years before the PH-type or HH-type onset and because indomethacin had no effect on the migraine-type headaches. This case is particularly notable because the patient presented with both PH-type and HH-type headaches, two distinct headache disorders that are responsive to indomethacin. The coexistence of PH and HH in a single patient appears to be exceedingly rare, with only limited documentation in the literature. We suspect that the patient does have both disorders, given the different clinical descriptions. However, given the onset of both headache types in the same year and the indomethacin responsiveness of both headaches, we cannot rule out a single disorder. Secondary PH-type and HH-type headaches have been described, and because of this, a recommended work-up for PH includes a brain MRI, vessel imaging of the head and neck, imaging of the apex of the lung, and pituitary function testing. The work-up for hypnic headache includes a brain MRI, erythrocyte sedimentation rate, C-reactive protein, polysomnography, and 24-hour blood pressure monitoring [11-14]. Our work-up focused on PH more than HH, and this work-up did show a possible nonfunctioning pituitary adenoma. PH-like headaches have been described in patients with pituitary adenomas. While the most common of a small sample appears to be prolactinoma, to our knowledge, nonfunctioning adenomas (which our patient may have) have been described not for PH but for cluster headache [12,15-16]. However, the typical presentation of these secondary headaches appears to be a single headache type.

Management for PH centers around an indomethacin trial, starting with 25 mg orally three times daily and increasing, if necessary, to 75 mg three times daily, along with a gastroprotectant (typically a proton pump inhibitor) [3,17]. After the initial treatment, patients can often reduce the maintenance dose needed to remain headache-free. For example, our patient initially used 25 mg twice daily but later needed only 25 mg once daily [1,18]. Unfortunately, indomethacin is poorly tolerated and can result in gastrointestinal, liver, or kidney issues [18]. In patients who cannot tolerate indomethacin, neuromodulation through noninvasive vagus nerve stimulation has been explored as an adjunctive therapy [3]. Cyclo-oxygenase-2 (COX-2) inhibitors like celecoxib, along with topiramate and melatonin, may also be effective in treating paroxysmal hemicrania [3,19].

Indomethacin-responsive headaches include PH, HH, hemicrania continua, primary cough headache, primary exercise headache, primary headache associated with sexual activity, and primary stabbing headache [6,18]. These conditions are characterized by their significant improvement with indomethacin, a nonsteroidal anti-inflammatory drug (NSAID) that inhibits cyclo-oxygenase-1 (COX-1) more selectively than most other NSAIDs [20]. The limited effectiveness of other NSAIDs in treating indomethacin-responsive headaches suggests a distinct underlying mechanism of indomethacin, such as the reduction of intracranial pressure or the reduction of nitric oxide-induced nociception [6,18]. Despite its effectiveness, the exact mechanism behind indomethacin’s action in these headache disorders remains unclear, as does its ineffectiveness in similar disorders such as cluster headache, SUNCT, and SUNA.

## Conclusions

This case highlights the rarity and diagnostic complexity of co-morbid indomethacin-responsive headaches, specifically paroxysmal hemicrania and hypnic headache. It underscores the effectiveness of indomethacin as both a diagnostic and therapeutic tool, despite its associated side effects. These findings emphasize the need for further research into the underlying pathophysiology of these disorders and the development of safer, long-term treatment strategies. Future studies should aim to address gaps in diagnostic testing and focus on optimizing management for patients with similar headache syndromes.
